# Bibliographical

**Published:** 1894-05

**Authors:** 


### EDITORIAL.

### Bibliographical.

ETIOLOGY OF OSSEOUS DEFORMITIES OF THE HEAD, FACE, JAWS, AND TEETH. By Eugene S. Talbot, M.D., D.D.S., Author of "Irregularities of the Teeth, and their Treatment." Author of "Chart of Typical Forms of Constitutional Irregularities of the Teeth," etc., etc. Third Edition, Revised and Enlarged with 461 Illustrations, 422 of which are original. This Work just issued from the Press of The W. T. Keener Company, Chicago.

This is a most valuable work; the outcome of years of study and patient investigation. In its preparation the Author has used not only that which he could gain by his own assiduous persevering effort in years of study and investigation, but he has received the aid and co-operation of others who have been engaged in the same line of work for a long period of time. It covers a broad field and is embraced in thirty-nine chapters, in which are presented almost every phase of the great subject with which it deals. It begins with a Historical Sketch of Theories Regarding the Etiology of Irregularities of the Maxillæ and Teeth; Changes in Climate; Intermixture of Races; Hereditary Influence; Development of the Cranium and Face; Development of the Jaws; Development of the Vault; Development of the Alveolar Process; Developmental Neuroses; Neurotics; Genius; Idiocy; Consanguinity of Parents; Intemperance; Maternal Impressions; City versus Country Life, etc., etc. These and other subjects are presented in a very clear and forcible manner. It is a work that may be studied with very great profit, not only by the dental practitioner and student, but by the physician and surgeon as well. We know of no other source where the same information may be so compactly detailed and clearly presented for the general student.

The illustrations, the larger proportion of which are original, are finely executed, and clearly present the points for which they were intended. We can not here attempt to give a review or criticism of the work, but simply desire with emphasis to call attention to its great value and many excellencies for the student in this line of investigation. We advise all our professional brethren to secure the work, and each one thoroughly review and criticise if need be for himself.

